# Seroreactivity to *Dirofilaria* antigens in people from different areas of Serbia

**DOI:** 10.1186/1471-2334-14-68

**Published:** 2014-02-08

**Authors:** Suzana A Tasić-Otašević, Simona V Gabrielli, Aleksandar V Tasić, Nataša L MiladinovićTasić, Jovana T Kostić, Aleksandra M Ignjatović, Lidija D Popović Dragonjić, Zoran G Milošević, Valentina S Arsić-Arsenijević, Gabriella A Cancrini

**Affiliations:** 1 Department of Microbiology and Immunology, Faculty of Medicine, University of Nis, 81, Bul. Dr. Zorana Djindjica, 18000 Nis, Serbia; 2 Center of Microbiology and Parasitology, Institute of Public health Nis, 50, Bul. Dr. Zorana Djindjica, 18000 Nis, Serbia; 3 Department of Public Health and Infectious Diseases, “Sapienza” University of Rome, Piazza le Aldo Moro 5, 00185 Rome, Italy; 4 Department of Medical Statistics, Faculty of Medicine, University of Nis, 81, Bulvd Dr. Zorana Djindjica, 18000 Nis, Serbia; 5 Faculty of Medicine, Department of Epidemiology and Infectology, Clinical Centar of Niš, Clinic for Infectious Disease, University of Niš, Bull Dr Zorana Djindjica 81, 18000 Nis, Serbia; 6 Department of Microbiology and Immunology, Faculty of Medicine, University of Belgrade, 1, Dr. Subotica, 11000 Belgrade, Serbia

**Keywords:** Human dirofilariosis, Sero-reactivity, Epidemiology, *Dirofilaria immitis*, *Dirofilaria repens*

## Abstract

**Background:**

The Northern part of Serbia is hyperendemic-endemic for canine dirofilarioses. Considering this fact, many human dirofilarial infections could be expected, however only about 30 cases in Serbia have been described until today. Aims of this survey were to assess the people reactivity to the antigens of *D. repens* and *D. immitis* and to identify risk factors for the contact exposure*.*

**Methods:**

Investigation included sera taken from 297 people (179 women and 118 men) living in different areas of Serbia (Pančevo, Novi Sad, Zaječar, Leskovac, Vranje, Niš, Pirot). Sera were analysed by means of two indirect enzyme-linked immunosorbent (ELISA) home-designed that use as antigens adult somatic/metabolic polyproteins of *D. repens* (DR) and *D. immitis* (DI), respectively. The results were elaborated using the statistical method of descriptive and quantitative analysis.

**Results:**

Significant differences by area in the reactivity of human sera to dirofilarial antigens were not observed (p = 0.056). A high seroreactivity was demonstrated in people from the towns of northern Serbia (Pančevo = 27,1%; Novi Sad = 16,3%), as well as in people from Zaječar (eastern Serbia = 15,8%) and Vranje (southern Serbia = 15,1%). No differences were evidenced between people reactivity to polyproteins of the two dirofilarial species, nor differences related to the gender of examinees. Factor risks evidenced were: i) place of residence; ii) spending work time outdoors during the mosquito season; iii) spending time outdoors and nearby rivers, lakes, swamps or canals; unespectedly, iv) cat owning.

**Conclusion:**

The findings emerging from this investigation indicate that clinicians and public health authorities should pay greater attention to this zoonosis. Continuing education and training of physicians will greatly contribute to the knowledge of the actual impact of filarial worms on animal and public health, and allow for the planning of suitable measures to prevent the infections.

## Background

*Dirofilaria immitis (D. immitis)* and *D. repens* are filarial nematodes that affect domestic and wild carnivores living in tropical and temperate regions of the World (the first one) and only in that of the Old World (the second). People are occasional and not fully suitable hosts of these parasites that, however, are being increasingly detected in subcutaneous or ocular tissues and, by accident, in asymptomatic more internal locations [1-4]. Considering the fact that only superficial infections can be easily detected, the number of people involved is surely more significant than believed and, therefore, this zoonosis deserves due attention by public health services.

Dirofilariae are mosquito-transmitted among animals and from animals to humans, after mosquito bloodsucking, when infective larvae L3 penetrate into the skin. The L3s, which in animals become adult worms producing in peripheral blood circulating microfilariae, in humans reach only seldom the adult stage; moreover, only exceptionally adult worms meet, mate and yield microfilariae [5,6], infectious for mosquitoes and useful for diagnostic purposes. Therefore, the abortive infections, in which the nematode size ranges from 1 mm and few centimeters, are difficult to be suspected, unless clinicians developed a consideration for these infections. The clinical implication is that pulmonary and, less commonly, subcutaneous lesions are initially misidentified as malignant tumors, requiring invasive investigations and surgery before the correct diagnosis is made. Parasite identification is usually performed on the basis of morphological study of the removed specimen and, more recently, following multilocus genetic analysis of gene-enzyme systems or molecular assays [7-9]. The latter diagnostic tool allows the identification of dirofilarial species also in specimens significantly damaged by the immunological reaction of the host and on minimal amounts of biopsy material, even if recovered from embedded sections [10,11]. However, the lesion has to be found and surgical removal of the worm is always necessary. These constrains made difficult to define the actual impact of the infection; however, since the penetration of active larvae is followed by a considerable antibody response, an alternative to parasitological/biochemical/molecular analyses of the biopsy material could be a non-invasive serology. At present, there are no commercial kits to diagnose human infections; however, home-made indirect enzyme-linked-immunosorbent assays (ELISAs) expressly designed can be applied.

The Northern part of Serbia is hyperendemic-endemic for canine dirofilarioses, as shown by researches that, in some territories, detected infection rates reaching over 60% [12,13]. By this epidemiological evidence in dogs, many cases of human infection should be expected, but instead, only about 30 cases have been reported to date [3,14]. This apparent disagreement urged us to investigate the actual frequency of infectious contacts between the two filarial parasites and human population, and to analyze possible risk factors for the infection, which would be useful in planning the appropriate control strategies.

## Methods

The study involved 297 people living in different areas of Serbia (Pančevo, Novi Sad, Zaječar, Leskovac, Vranje, Niš, Pirot) included between 42,33°-45,15°N and 19,50°-22,17°E.

At the beginning of research, it was expected that infection prevalence would be 10%. Based on this assumed value, considering an infinite population size, 5% maximum error desired, the minimal sample size of 139 would be needed. Afterwards, this sample size was doubled to overcome the event of possible higher prevalence in some areas. Calculation of sample size was performed by an online calculator (http://sampsize.sourceforge.net/iface/index.html). The Ethic Committee of the University of Nis, Faculty of Medicine (decision No 01-4608-2/2009) approved this research. The agreed number of participants was chosen from the general list of patients with application of random number table. All the patients gave written consent and filled in a semi-structured questionnaire. The patients were all adults.

Relevant data [age, place of habitation (urban or rural region) and of residence (apartment or house), education level, occupation (in the open or not) and way of spending spare time (outside or not and, in detail, nearby rivers, lakes, swamps, canals or not), keeping dogs or cats, usage of mechanical (nets) or chemical (insecticides and repellents against mosquitoes) preventive measures were collected from each subject who filled a semi-structured inquiry form.

### Detection of specific antibodies

Sera were analysed by means of two home-made ELISA assays that use as antigens somatic (SA) and excretory/secretory (E/S) polyproteins of adult *D. immitis* (DI) and *D. repens* (DR) [15], formerly applied also to study the reactivity to dirofilarial antigens in Serbian dogs [13]. Antigenic complexes were obtained as previously described [16]. Briefly, worms obtained by necropsy of naturally infected dogs were washed, macerated and sonicated (three cycles of 70 kHz, 30 s) in sterile saline solution. The homogenate was centrifuged at 16000 × g for 30 minutes. The supernatant was dialyzed against 0.01 M PBS, pH 7.2. The protein concentration was measured [17] and adjusted to 4.5 μg/μL and 10 μg/μL final solution for SA-DI and SA-DR, respectively. All procedures were carried out at 4°C. E/S antigens were prepared from worms cultured at 37°C for several days as described by Santamaria et al. [18]. The protein concentration was measured [17] and adjusted to 2 μg/μL and 5 μg/μL final solution for E/S-DI and E/S-DR, respectively. Antigens were stored at -20°C until use.

A 1:1 pool of SA and E/S antigens from *D. immitis* and *D. repens* were used for each assay, and the final antigen concentration for microplate coating was 0.8 μg/μL. Sera were tested in solid-phase ELISA at dilution 1:50. The anti-human-IgG-peroxidase conjugate to horseradish peroxidase (Dako, Glostrup, Denmark) was used as a secondary antibody at 1:5000 dilution. The optical density (OD) was measured at 492 nm (Easy-Reader, Bio Rad). Optimal OD cut-off value was 0.5 for both filarial species (OD arithmetical mean plus three standard deviations obtained for 10 children 5-10 years-old living in mountainous areas dirofilaria*-*free). Controls used were the aforementioned negative sera stored as negative, and sera from people with documented infection by *D. repens* or *D. immitis*.

### Statistical analyses

Data are presented as mean and standard deviation, median and interquartile range, or percent frequency, with p = 0.05 indicating statistical significance. Comparisons between groups were made using the Mann-Whitney test and Fisher exact test (the chi-square test for more than two groups) for nominal variables and for non-parametric data, respectively. The individual examinee data were analyzed as a dependent variable by the logistic regression model, using both *D. repens* and *D. immitis* status. Statistical analysis of data was performed using the R 2.15.3 software (R Foundation for Statistical Computing, Vienna, Austria).

## Results

This prospective study included 118 males and 179 females whose mean age was 52,77 ± 15,72. The baseline characteristics of the study population and his reactivity to the two-dirofilarial nematodes are summarized in Table 1.

**Table 1:** Seroreactivity to D. repens and D. immitis antigens detected in examinees (percent of positives and negatives)

		***D. repens *** **antigens**			***D. immitis *** **antigens**			**Overall seroreactivity**		
		**Positive**	**Negative**	**p**	**Positive**	**Negative**	**p**	**Positive**	**Negative**	**p**
		**n=29**	**n=268**		**n=24**	**n=273**		**n=46**	**n=251**	
**A**#		48.3±19.66	53.3±15.6	0.344	55.8±17.2	52.5±15.9	0.226	51.6±19.3	53.0±15.4	0.658
**B**	M	31.0	40.7	0.314	41.7	39.6	0.840	37.0	40.2	0.676
	F	69.0	59.3		58.3	60.4		63.0	59.8	
**C**	U	34.5	35.1	0.949	37.5	34.8	0.790	34.8	35.0	0.971
	R	65.5	64.9		62.5	65.2		65.2	65.0	
**D**	A	72.4	83.6	0.133	70.8	83.5	0.117	71.7	84.5	0.037
	H	27.6	16.4		29.2	16.5		28.3	15.5	
**E**	1	44.8	48.9	0.492	54.2	48.0	0.824	50.0	48.2	0.676
	2	41.4	43.6		37.5	44.0		39.1	44.2	
	3	13.8	7.5		8.3	8.0		10.9	7.6	
**F**	0	34.5	44.8	0.289	58.3	42.5	0.134	45.6	43.4	0.780
	1	65.5	55.5		41.7	57.5		54.4	56.6	
**G**	0	17.2	27.6	0.230	24.9	45.8	0.026	25.9	30.4	0.522
	1	82.8	72.4		75.1	54.2		74.1	69.6	

# age (A), gender (B: M = male, F = female), place of habitation (C: U = urban, R = rural), and residence (D: A = apartment, H = house), education level (E: 1-elementary school, 2-high school, 3- college or university), dog keeping (F: 0 = not, 1 = yes), cat keeping (G: 0 = not, 1 = yes).

A total of 46 people (17 men and 29 women) were reactive to *Dirofilaria* antigens, accounting for an overall positivity of 15.4% (95% CI 11.8-20.0%). Specific antibodies against DR polyproteins were found in 29 (9.7%, 95% CI 6.9-13.7%) subjects, and against that of *D. immitis* in 24 (8.1%, 95% CI 5.5-11.7%) individuals; seven of them (2.3%, 95% CI 1.2-4.8%) had specific antibodies to both species.

The different reactivity to *D. repens* and *D. immitis* found was not significant (p = 0.551), nor was significant that evidenced between people reactive and non-reactive to DR antigens regarding all considered risk factors. On the contrary, cat owners were significantly more reactive to DI antigens (p = 0.026) and the overall serological results depended on the “place of residence” variable (people who are living in the house, not in apartment, were more reactive to both dirofilarial antigens; p = 0.037). The univariate logistic regression analysis confirmed the non-association between reactivity to DR polyproteins and all baseline characteristics of the study population, and got same significant differences: positive serology to DI antigens was associated with cat-owners OR = 2.55 (95% CI 1.121-7.381, p = 0.031), overall reactivity with the place of residence OR = 2.141 (95% CI 1.035-4.430, p = 0.040).

Moreover, no differences were found among the different areas (p = 0.056), but a higher seroreactivity was found in Pančevo (27.1%), Novi Sad (16.3%), Zaječar (15.8%) and Vranje (15.1%), whereas the lowest figure was found in Niš (5.3%) (Table 2).

**Table 2:** Seroreactivity to dirofilarial antigens in people living in Serbia, by areas

** Area**	**Geographical coordinates**	**Positive examines N**
		**(%, 95% CI)**
Pančevo	44.86°N; 20.64°E	16/59
		(27.1%, 17.4-39.6%)
Novi Sad	45.15°N; 19.50°E	7/43
		(16.3%, 8.1-29.9%)
Zaječar	43.54°N; 22.17°E	3/19
		(15.8%, 5.5-37.6%)
Leskovac	42.59°N; 21.56°E	3/35
		(8.6%, 2.9-22.4%)
Vranje	42.33°N; 21.53°E	14/93
		(15.1%, 9.2-23.7%)
Niš	43.19°N; 21.54°E	1/19
		(5.3%, 0.1-24.6%)
Pirot	43.09°N; 21.54°E	2/30
		(6.7%, 1.8-21.3%)
Total		46/297
		(15.4%, 11.8-20.0%)

Finally, the analysis of the inquiry showed further significant differences between subjects reactive and non-reactive to: i) DR antigens, associated to work time conditions (spending time outdoors during the mosquito season; p = 0.048); ii) DI antigens, associated with both work time conditions (spending time outdoors; p = 0.049) and spending time outdoors near by rivers, lakes, swamps or canals (p = 0.036).

## Discussion

In Serbia, during the thirties of the last century, the southern part of the country was a hyperendemic area for *D. repens* infection in dogs. At present this epizootiological status has been observed in the northern part of the country, and in the mean time in the southern one there has been a significant decrease of dirofilariosis prevalence [19]. This trend is expected, as it is assumed the northern border of endemic zones moves up to the north of the continent due to increasing temperature [20], not suitable for the mosquitoes species that act as vectors [21]. Therefore, the epidemiology of animal and human dirofilariosis, as all vector-borne infections, is changing and requires continuous updating. Immunological tests, if sufficiently sensitive and specific, facilitate such epidemiological investigation and play an important role in assessing the actual frequency of the infectious contacts between the two filarial nematodes and human population. Unfortunately, commercial kits are not yet available to better investigate the issue, and therefore we carried out a serological survey by means of home-made ELISAs formerly designed with polypeptides isolated from the two dirofilarial species. To solve the specificity and sensibility troubles related to the home-made assay, we employed somatic antigens, which allow high sensitivity, associated with E/S antigens, which assure higher specificity. In fact, such tests were been applied with satisfactory results in serological surveys carried out in Italian and Spanish endemic areas and, in association with morphology and molecular tools, to characterize several cases of documented human infection [10,22]. However, their application evidenced that the sensibility, which is obviously affected by immunological reactivity of the subject, depends also on the time elapsed between infection and blood sampling. Moreover, it has been observed that ocular/peri-ocular locations sometime fail to be detected [10,22-24]. On the contrary, polyproteins here used were found to give very high specificity, not only against sera from patients with *Toxocara canis, Trichinella spiralis*, *Strongyloides stercoralis*, *Echinococcus granulosus* and *Fasciola hepatica* but even against sera from patients with tropical filarioses [15,22]. Therefore, the results here presented risk to be an underestimation of the actual prevalence.

With this premise and despite the facts that the numbers of available sera for each area were a bit different, and the mean age of examined people was rather high, our preliminary findings can be used for a general evaluation of the sanitary problem and to underline that a part of Serbian population is a risk of infection by dirofilarial species.

Human reactivity to antigens of dirofilarial worms documents a noticeable possibility of the parasite transmission from animals to humans, matching the prevalence of *D. repens* and *D. immitis* detected in the dog population [13]. Although all the described cases of human dirofilariosis in Serbia recognized *D. repens* as the etiological agent, our findings indicated that *D. immitis* too came in contact with humans, with about the same frequency as *D. repens* (8.1% vs 9.7%). This finding is not surprising, as it is in agreement with observations made in all investigated countries where the two-dirofilarial species are present [25,26]. Perhaps *D. immitis* induces a strong immune response of the host that blocks the worm development at the third larval stage, very small and difficult to be detected or, more simply, that the preferred superficial location of *D. repens* facilitates its detection. Further possible explanations include different degree of anthropophilic behavior of the mosquito vectors in the studied areas [27,28], and the immunocompetition between the two dirofilariae as suggested by laboratory infections [29].

Preliminary serological results here reported are in general agreement with available literature data that describes most cases of human infection in people living in the area of Belgrade and in Vojvodina, and one case in a patient from the city of Zaječar [14], where we are now finding a high reactivity (15,8%). This survey proved a high reactivity to dirofilarial antigens in people living in Pančevo (27.1%), Novi Sad (16.3%) and Vranje (15.1%). Findings relative to the first two areas match the studied infection rates in dogs (Pančevo = 35.6%; NoviSad = 28.9%) [13], whereas the results concerning Vranje were expected as the latest cases of superficial dirofilariosis due to *D. repens* were diagnosed in two patients from this city of Southern Serbia [3]. A lower reactivity was found in Leskovac (8.6%) and Pirot (6.7%) and the lowest was proven in Niš (5.3%). These findings are also entirely congruent with the results of surveys we carried out on dogs [30]*.*

The association between positive serology with spending a lot of time outdoors in occupational exposure, during a mosquito season, and nearby the rivers, lakes, swamps or canals is quite understandable. Indeed, additional exposure to by-day biting vectors (like *Stegomya albopicta*) significantly increases the possibility of transmission of dirofilariosis. The apparent uselessness of mechanical preventive measures, insecticides and repellents against mosquitoes when subjects are spending time outdoors can be explained by occasional use of such protection tools or by the usage of inadequate mechanical or chemical means. Finally, the different relevance of owning cats or dogs in order to become seroreactive to *D. immitis* here found needs further investigations. We can only guess that it could be related to the epidemiological relevance of mosquito vector species biting by day (like *S. albopicta*), when the cat (that usually is sleeping) is an easy source of blood. However, data on dirofilariosis prevalence in these pets are not available.

The noticeable seroreactivity evidenced and the absence of a corresponding number of proven infections can be attributed to well-known global drivers [4] and, respectively, to the fact that most of them are asymptomatic, or unsuspected by physicians (unaware of this parasitic infection), or unreported. Therefore, available data on human dirofilarioses in Serbia, like everywhere, represent only the tip of an iceberg, whose actual size is still unknown.

## Conclusion

The significant reactivity to dirofilarial antigens evidenced in people, in a relatively little sample of population living in different areas of the country, suggests that more extensive and detailed investigations coupled with continuing education and training of physicians are needed. Likely, the increased attention paid in recent years to the dirofilariosis issue from the “one health” perspective is creating the basis for better define the impact of dirofilarioses on humans and to plan suitable measures to prevent these often neglected infections.

## Competing interests

The authors declare that they have no competing interests.

## Authors’ contributions

STO: designed research, carried out the literature review, drafted the manuscript, JK: contributed substantially including translation to the literature review. GC, SG: carried out the home-made immunoassays, helped draft the manuscript and contributed significantly to its design and revision. AI, ZM: carried out the statistical analyses. NMT, LPD, VAA: collected, assembled the data of examinees and drafted the manuscript. AT: revised the manuscript and contributed significantly to its design. All authors read and approved the final manuscript.

## Pre-publication history

The pre-publication history for this paper can be accessed here:

http://www.biomedcentral.com/1471-2334/14/68/prepub

## References

[B1] Pampiglione S Rivasi F Gustinelli A Dirofilarial human cases in the old world, attributed to dirofilaria immitis: a critical analysis Histopathology 2009 14 2 192 204 10.1111/j.1365-2559.2008.03197_a.x 19207944

[B2] Avellis FO Kramer LH Mora P Bartolino A Benedetti P Rivasi F A case of human conjunctival dirofilariosis by Dirofilaria immitis in Italy Vector Borne Zoonotic Dis 2011 14 4 451 452 10.1089/vbz.2010.0067 20846011

[B3] Tasic S Stoiljkovic N Miladinovic-Tasic N Tasic A Mihailovic D Rossi L Gabrielli S Cancrini G SubcutaneousdirofilariosisinSouth-EastSerbia–casereport Zoonoses public health 2011 14 5 318 322 10.1111/j.1863-2378.2010.01379.x 21740534

[B4] Simón F Siles-Lucas M Morchón R González-Miguel J Mellado I Carretón E Montoya-Alonso JA Human and animal dirofilariasis: the emergence of a zoonotic mosaic Clin Microbiol Rev 2012 14 3 507 544 10.1128/CMR.00012-12 10.1128/CMR.00012-12 22763636 PMC3416488

[B5] Nozais JP Bain O Gentilini M A case of subcutaneous dirofilaria (Nochtiella) repens with microfilaremia originating in Corsica Bull Soc pathol exot 1994 14 3 183 185 7827520

[B6] Kucsera I Danka J Fok E Salomváry B Orosz E Human dirofilaria repens infection in Hungary: past and nowadays 46 Days o fPreventive Medicine: 2012 2012 Nis, Serbia: Public Health Institute Nis, University of Nis, Faculty of Medicine

[B7] Cancrini G Mattiucci S D’Amelio S Genchi C Coluzzi M Genetic characterization of Dirofilaria repens and D.immitis by electrophoretic analysis of gene-enzyme systems Parassitologia 1989 14 2–3 189 196 2486999

[B8] Favia G Lanfrancotti A DellaTorre A Cancrini G Coluzzi M Polymerase chain reaction-identification of Dirofilaria repens and Dirofilaria immitis Parasitology 1996 14 Pt6 567 571 8939053 10.1017/s0031182000067615

[B9] Favia G Cancrini G Ricci I Bazzocchi C Magi M Pietrobelli M Genchi C Bandi C 5S ribosomal spacersequences of some filarial parasites: comparative analysis and diagnostic applications Mol Cell Probes 2000 14 5 285 290 10.1006/mcpr.2000.0317 11040091

[B10] Cancrini G Prieto G Favia G Giannetto S Tringali R Pietrobelli M Simon F Serological assays on eight cases of human dirofilariasis identified by morphology and DNA diagnostics Ann Trop Med Parasitol 1999 14 2 147 152 10.1080/00034989958627 10474639

[B11] Gabrielli S Tasic S Magi M Cancrini G Morchón R, Simón F, Montoya JA Molecular diagnosis of human dirofilariosis from paraffin- embedded nodules Second EuropeanDirofilaria days: 2009 2009 Salamanca, Spain: Morchón R, Simón F, Montoya JA, Genchi C 216 Genchi C.ISBN-13: 978-84-692-3583-6

[B12] Tasic A Rossi L Tasic S Miladinovic-Tasic N Ilic T Dimitrijevic S Survey of canine dirofilariasis in Vojvodina, Serbia Parasitol Res 2008 14 6 1297 1302 10.1007/s00436-008-1132-z 18712415

[B13] Tasic A Tasic-Otasevic S Gabrielli S Miladinovic-Tasic N Ignjatovic A Dordevic J Dimitrijevic S Cancrini G Canine dirofilaria infections in two uninvestigated areas of Serbia: epidemiological and genetic aspects Vector Borne Zoonotic Dis 2012 14 12 1031 1035 10.1089/vbz.2011.0949 23127188 PMC3525891

[B14] Dzamic AM Colovic IV Arsic-Arsenijevic VS Stepanovic S Boricic I Dzamic Z Mitrovic SM Rasic DM Stefanovic I Latkovic Z Human Dirofilaria repens infection in Serbia J Helminthol 2009 14 2 129 137 10.1017/S0022149X09341346 19379543

[B15] Cancrini G Montoya MN Prieto G Bornay-Linares FJ Simon F, Genchi C Current advances in experimental diagnosis of human and animal dirofilariosis Heartworm infection in humans and animals 2001 103 120

[B16] Prieto C Venco L Simon F Genchi C Feline heartworm (Dirofilaria immitis) infection: detection of specific IgG for the diagnosis of occult infections Vet Parasitol 1997 14 4 209 217 10.1016/S0304-4017(97)00008-3 9211646

[B17] Bradford MM A rapid and sensitive method for the quantitation of microgram quantities of protein utilizing the principle of protein-Dye binding Anal Biochem 1976 14 248 254 10.1016/0003-2697(76)90527-3 942051

[B18] Santamaria B Cordero M Muro A Simon F Evaluation of Dirofilaria immitis excretory/secretory products for seroepidemiological studies on human dirofilariosis Parasite 1995 14 269 273 8520802 10.1051/parasite/1995023269

[B19] Simin SLV Tasić A Spasojević-Kostić L Mitić V First report of Dirofilaria repens in dogs from Southest Serbia7th Balkan Congress for Microbiology: 2011 2011 Belgrade, Serbia: Serbian Society of Microbiology

[B20] Genchi C Rinaldi L Cascone C Mortarino M Cringoli G Is heart worm disease really spreading in Europe? Vet Parasitol 2005 14 2–3 137 148 15885913 10.1016/j.vetpar.2005.04.009

[B21] Cancrini G Gabrielli S Morchón R, Simón F, Montoya JA Global warming: effects on abundance and distribution of the Dirofilaria natural vectors2nd European Dirofilaria days: 2009 2009 Salamanca, Spain: Morchón R, Simón F, Montoya JA, Genchi C 98 106 Genchi C.ISBN-13: 978-84-692-3583-6

[B22] Prieto G Cancrini G Muro A Genchi C Simon Martin F Seroepidemiology of Dirofilaria immitis and Dirofilaria repens in humans from three areas of Southern Europe Res Rev Parasitol 2000 14 3/4 4

[B23] Simon F Prieto G Muro A Cancrini G Cordero M Genchi C Human humoral immune response to Dirofilaria species Parassitologia 1997 14 4 397 400 9802100

[B24] Cancrini G Gabrielli S Tasić S Miladinović-Tasić N Tasić A Đorđević J Peri-orbital human dirofilariosis: a pitfall for experimental ELISA Acta Facultatis Medicae Naissensis 2010 14 4 185 188

[B25] Pampiglione S Rivasi F Human dirofilariasis due to Dirofilaria (Nochtiella) repens: an update of world literature from 1995 to 2000 Parassitologia 2000 14 3–4 231 254 11686084

[B26] Genchi C Kramer LH Rivasi F Dirofilarial infections in Europe Vector Borne Zoonotic Dis 2011 14 10 1307 1317 10.1089/vbz.2010.0247 21417922

[B27] Cancrini G Della Torre A Coluzzi M Different probabilities of transmission of Dirofilaria immitis and D.repens by Culex pipiens VIth European Meeting of Parasitology 1992 90

[B28] Cancrini G Gabrielli S Genchi C, Rinaldi L, Cringoli G Vectors of Dirofilaria nematodes: biology, behaviour and host/parasite relationships Dirofilaria immitis and D repens in dog and cat and human infections 2007 48 58

[B29] Genchi C Basano SF Bandi C DiSacco B Venco L Vezzoni A Cancrini G Factors influencing the spread of heartworms in Italy: interaction between Dirofilaria immitis and Dirofilaria repens Heartworm Symposium 1995 American Heartworm Society 65 71

[B30] Tasić A Tasic S Miladinović-Tasić N Zdravković D Djordjević J Prevalence of Dirofilaria repens- cause of zoonosis in dogs Acta Facultatis Medica eNaissensis 2007 14 2 72 75

